# Structure and function of the Arctic and Antarctic marine microbiota as revealed by metagenomics

**DOI:** 10.1186/s40168-020-00826-9

**Published:** 2020-04-02

**Authors:** Weipeng Zhang, Shunan Cao, Wei Ding, Meng Wang, Shen Fan, Bo Yang, Andrew Mcminn, Min Wang, Bin-bin Xie, Qi-Long Qin, Xiu-Lan Chen, Jianfeng He, Yu-Zhong Zhang

**Affiliations:** 1 College of Marine Life Science, Frontiers Science Center for Deep Ocean Multispheres and Earth System, Ocean University of China, Qingdao, 266003 China; 2 Laboratory for Marine Biology and Biotechnology, Pilot National Laboratory for Marine Science and Technology (Qingdao), Qingdao, 266373 China; 3 The Key Laboratory for Polar Science SOA, Polar Research Institute of China, Shanghai, 200136 China; 4 State Key Laboratory of Microbial Technology, Marine Biotechnology Research Center, Shandong University, Qingdao, 266237 China

**Keywords:** Global ocean microbiome, Arctic and Antarctic zones, Environmental adaptation

## Abstract

**Background:**

The Arctic and Antarctic are the two most geographically distant bioregions on earth. Recent sampling efforts and following metagenomics have shed light on the global ocean microbial diversity and function, yet the microbiota of polar regions has not been included in such global analyses.

**Results:**

Here a metagenomic study of seawater samples (*n* = 60) collected from different depths at 28 locations in the Arctic and Antarctic zones was performed, together with metagenomes from the *Tara* Oceans. More than 7500 (19%) polar seawater-derived operational taxonomic units could not be identified in the *Tara* Oceans datasets, and more than 3,900,000 protein-coding gene orthologs had no hits in the Ocean Microbial Reference Gene Catalog. Analysis of 214 metagenome assembled genomes (MAGs) recovered from the polar seawater microbiomes, revealed strains that are prevalent in the polar regions while nearly undetectable in temperate seawater. Metabolic pathway reconstruction for these microbes suggested versatility for saccharide and lipids biosynthesis, nitrate and sulfate reduction, and CO_2_ fixation. Comparison between the Arctic and Antarctic microbiomes revealed that antibiotic resistance genes were enriched in the Arctic while functions like DNA recombination were enriched in the Antarctic.

**Conclusions:**

Our data highlight the occurrence of dominant and locally enriched microbes in the Arctic and Antarctic seawater with unique functional traits for environmental adaption, and provide a foundation for analyzing the global ocean microbiome in a more complete perspective.

Video abstract.

## Introduction

Recent advances in molecular ecological techniques, such as metagenomics, have enabled a more efficient pathway to address the taxonomic and functional composition of marine microbiota, including the free-living bacterioplankton [1–3], the sediment-dwelling microbes [4–6], the surface-attached biofilms [7–9], and the animal-associated symbionts [10–12]. Pursuing the goal characterizing the global seawater microbiota, the *Tara* Oceans expedition collected seawater samples from 68 locations, representing all main oceanic regions, and performed metagenomics to study the structure and function of the global ocean microbiota [1]. As a result, global ocean microbial diversity has been systematically investigated, and found to consist of more than 35,000 microbial “species” [1]. It was noted that depth is the major factor structuring the microbial community, explaining 73% of the variance in composition [1]. More importantly by constructing an ocean microbial reference gene catalog (OM-RGC), the *Tara* Oceans project identified ubiquitous genes that are enriched for functions including coenzyme, lipid, nucleotide, amino acids, and secondary metabolites transport [1]. However, one shortcoming of this holistic global ocean study is that no Arctic samples and only five Antarctic samples were included.

The Arctic and Antarctic are two of the most geographically distant bioregions on Earth. The two regions have similar physical-chemical characteristics; characterized by low temperature, low availablility of carbon sources, and extreme seasonality in light conditions [13, 14]. Attention has been paid to the changes caused by climate change in both of the polar regions, such as the record of water freshening [15, 16] and greenhouse gas emission [17, 18]. However, there are also differences; surface temperatures over much of the Arctic are continually increasing while at the moment major increases in Antarctic temperatures are limited to the Antarctic Peninsula area [19–21]; there is even record of increasing sea-ice coverage in the Antarctic based on satellite-derived observations from 1979 to 2015 [22].

Over the past decades there have been a number of attempts to understand the distribution of microbioal taxa in the polar oceans. Galand et al. [23] focused on rare taxa in Arctic seawater and highlighted the role of ecological processes such as selection and extinction on their biogeography. Ghiglione et al. [24] studied the taxonomic structures of microbiota in Arctic and Antarctic water and highlighted the difference between polar and non-polar seawater microbiota, as 78% of operational taxonomic units (OTUs) were unique to the Southern Ocean and 70% were unique to the Arctic Ocean; consistently, the finding by Kleinteich et al. highlighted the pole-to-pole connections of the seawater microbiota [25]. It was also found that deep ocean communities differed less between polar and non-polar waters in comparison with the surface ocean communities [24]. By sampling marine epipelagic bacterial communities from the Arctic, Atlantic, Pacific, and Southern oceans, the study by Sul et al. [26] revealed latitudinal gradient in bacterial diversity.

Despite the previous efforts, a systematic understanding of the structure of polar microbiota and its place in a global context is lacking; more importantly, as most of the studies were based on 16S rRNA gene amplicon sequencing, functional characteristics linking microbial adaptation with the polar environment remains elusive. Thus, the aim of the present study is to address the following questions: (i) how does endemism in polar regions compare with elsewhere in the world; (ii) what are the specific functions of the polar microbiota, and how do these functions contribute to environmental adaptation. During the cruises to the Arctic and Antarctic, 60 samples were collected from 28 sites across multiple depths. The samples underwent metagenomic sequencing and were compared with data from the *Tara* Oceans project.

## Results and Discussion

### Structure and diversity of the polar microbiota

**Fig. 1:**
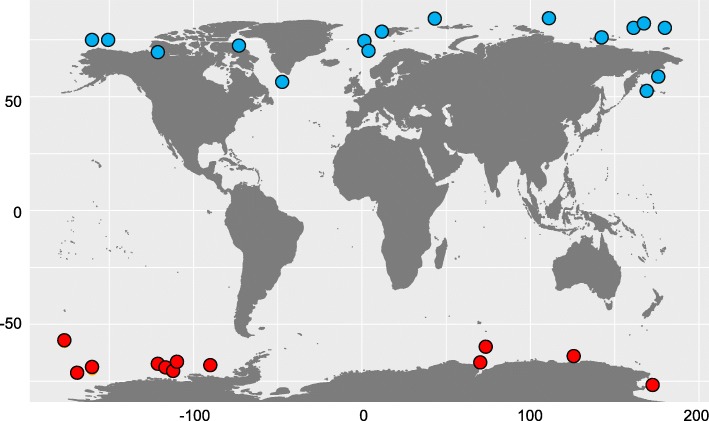
Geographic distribution of the 28 sampling locations of the Arctic (blue) and Antarctic (red) seawater

**Fig. 2:**
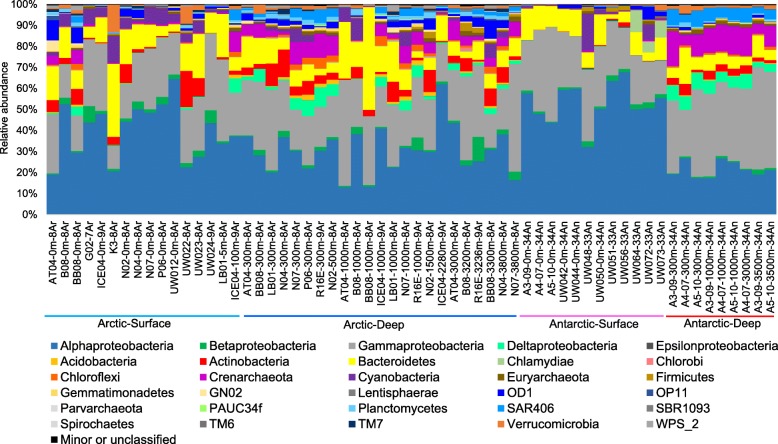
Phylum-level (class level for Proteobacteria) taxonomic structure of the 60 polar seawater metagenomes. Relative abundance of the phyla was calculated based on 16S miTag numbers. The top 30 phyla in terms of maximum relative abundance among the metagenomes are shown with all other phyla and the unclassified miTags are grouped together as “Minor or unclassified.” The four groups: Arctic-Surface (0-100 m), Arctic-Deep (200-4000 m), Antarctic-Surface (0-100 m), and Antarctic-Deep (200-4000 m)

Sampling locations are shown in Fig. 1. A total of 39 Arctic seawater and 21 Antarctic seawater samples were collected for this project. Based on the sample depth, the samples were further divided into four groups: Arctic-Surface (0-100 m), Arctic-Deep (200-4000 m), Antarctic-Surface (0-100 m), and Antarctic-Deep (200-4000 m). Correspondingly, 60 metagenomes were obtained after Illumina sequencing. 16S rRNA gene sequences were extracted from the metagenomes for analysis and are subsequently referred to as 16S miTags. Classification of OTUs at 97% similarity resulted in a total of 24,504 OTUs. Classification of OTUs at the phylum level (class level for Proteobacteria) (Fig. 2) revealed 90 taxa, with a dominance by Alphaproteobacteria and Gammaproteobacteria in most of the metagenomes, followed by Bacteroidetes. In addition, Deltaproteobacteria, Actinobacteria, Cyanobacteria, Crenacrchaeota, OD1 (Parcubacteria), and SAR406 (Marinimicrobia) showed high relative abundances in certain metagenomes. When the OTUs were classified at genus level (Additional file 1: Figure S1), 2,121 genera were obtained, dominated by an unnamed Pelagibacteraceae group. A total of 12.63 ± 0.06% of the reads could not be classified to genus level.

**Fig. 3:**
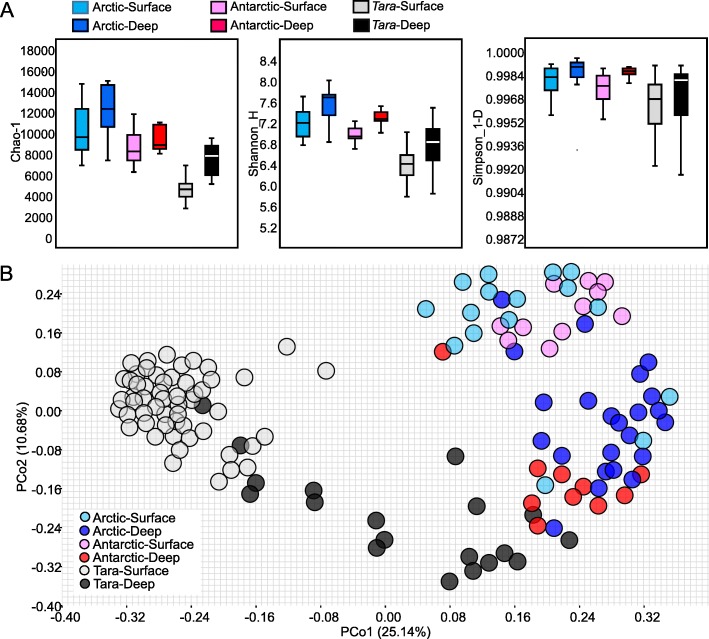
Alpha- and betadiversity analyses of the polar and *Tara* Oceans microbiomes. **a** Chao1, Shannon, and Simpson diversities were calculated based on miTags normalized to the same library size (10,000 sequences per metagenome). **b** Jaccard similarity of the microbial communities illustrated by principal coordinates analysis of the OTU matrix (item and tag numbers). This analysis was performed after normalizing the miTags data to the same library size (10,000 sequences per metagenome). The six groups: Arctic-Surface (0-100 m), Arctic-Deep (200-4000 m), Antarctic-Surface (0-100 m), Antarctic-Deep (200-4000 m), *Tara*-Surface (5 m), and *Tara*-Deep (100-1000 m)

To examine the similarity between the Arctic, Antarctic and the non-polar seawater microbial communities, 55 surface seawater (5 m) and 19 deep seawater metagenomes (100-1000 m) were downloaded from the *Tara* Oceans project [1] (http://ocean-microbiome.embl.de/companion.html) for comparison. Alpha-diversity analysis (Fig. 3a), based on rarefied 16S miTags (10,000 miTags per metagenome), revealed an overall higher Chao1 diversity, Shannon diversity, and Simpson diversity for the polar microbial communities; the alpha-diversities in the deep seawater samples tended to be higher than those in the surface seawater. Principal coordinates analysis (PCoA) (Fig. 3b) using rarefied 16S miTag data revealed clear separation of the polar from the non-polar seawater microbiota, especially for the surface microbiota, with PCo1 explaining 25.14% of the variability. Interestingly, the Arctic-Surface and the Antarctic-Surface seawater were clustered together, as were the Arctic-Deep and Antarctic-Deep seawater (Fig. 3b).

As found in previous studies based on 16S rRNA gene amplicon sequencing [23–26], the microbiota of Arctic and Antarctic seawater are more similar to each other than to seawater from other regions, and the dissimilarity between polar and temperate communities is more marked at the surface than in deeper water. Consistent with these findings, metagenomic analyses performed in the present study revealed a high similarity between microbiota in the two polar regions, which is likely to be attributed to environmental filtering (effect of environmental variables on community composition) and microbial dispersal, as the environmental conditions in the Arctic and the Antarctic are more similar to each than to the temperate regions. Higher diversity of microbes in deep polar seawater than in surface seawater was observed, implying that the deep waters are more of a reservoir of microbial species. Moreover, a higher similarity between the deep waters of temperate and polar area waters than between the surface waters of polar and temperate areas were found, which can be explained by the fact that surface communities have a high phylogenetic turnover rate [27] and minimal microbial connectivity by major currents [28], whereas the biogeography of deep water communities is largely controlled by ocean circulation [29].

### Taxonomic and functional specificity

**Fig. 4:**
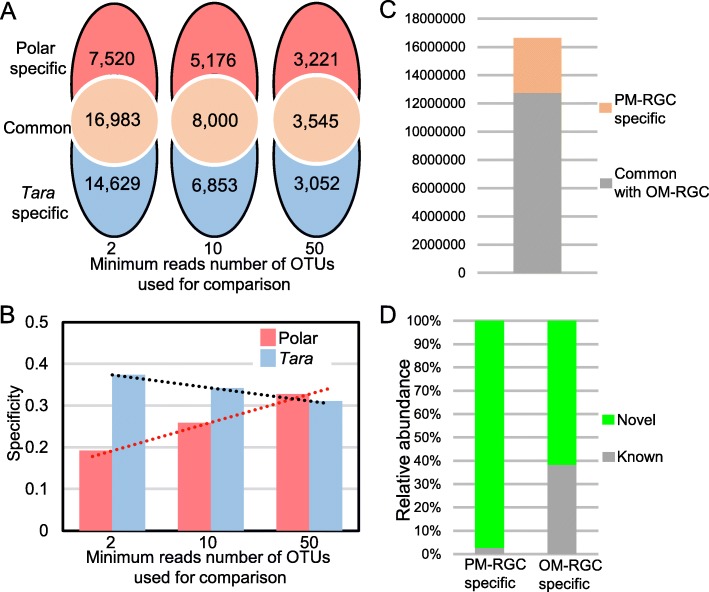
Comparison between the polar microbiomes and the *Tara* Oceans data. **a** Venn diagram showing the distribution of operational taxonomic units (OTUs) across the polar and the *Tara* miTags. The *Tara* miTags comprise 16S rRNA sequences extracted from 243 seawater metagenomes. Venn diagrams based on OTUs with more than 2, 10, 50 miTag numbers are shown. **b** The change of the taxonomic specificity along with the OTU abundances (indicated by the reads number of OTUs) included for comparison. **c** Establishment of a polar microbial reference gene catalog (PM-RGC) and comparison with the ocean microbial reference gene catalog (OM-RGC) established by the Tara Oceans study. PM-RGC specific gene orthologs are nonredundant genes that are present in the polar seawater metagenomes but were not detected in the OM-RGC. **d** BLASTp searching the NCBI-Nr database using the PM-RGC or OM-RGC specific gene catalog. Genes got hit in NCBI-Nr database are labeled as “known” while genes with no hit are labeled as “novel”

The 60 polar metagenomes were combined and the overlapping OTUs between all the polar seawater microbiota and all the *Tara* seawater microbiota (*n* = 243) were analyzed. The reads were mapped back to the OTUs; of the OTUs with more than 2 reads, 7520 were specific for the polar seawater; of the OTUs with more than 10 reads, 5176 were specific for the polar seawater; and of the OTUs with more than 50 reads, 3221 were specific for the polar seawater; this exceeded the number of OTUs specific for the *Tara* Oceans seawater (Fig. 4a). The specificity of the polar seawater (i.e., the ratio of polar-specific OTUs to the total number of OTUs present in the polar and non-polar seawater) consistently increased, whereas the specificity of the *Tara* Oceans seawater decreased with the number of minimum sequences possessed by the OTUs used for examination (Fig. 4b).

When the functions of the polar seawater microbiota were analyzed, a total of 16,638,499 orthologs were derived from the 60 metagenomic assemblies, resulting in a polar marine reference gene catalog (PM-RGC). In comparison with the OM-RGC using BLASTp, 3,903,052 (23.46%) of the PM-RGC orthologs were specific (*e* value > 1e-7 or similarity < 40%) (Fig. 4c). The orthologs specific for OM-RGC or PM-RGC were further searched against the NCBI-Nr database. It was found that more than 97% of the PM-RGC specific orthologs could not be identified (*e* value >1 e-7 or similarity < 40%) in the NCBI-Nr database, while about 61% of the OM-RGC specific orthologs had no hits (*e* value > 1e-7 or similarity < 40%) in NCBI-Nr (Fig. 4d).

**Fig. 5:**
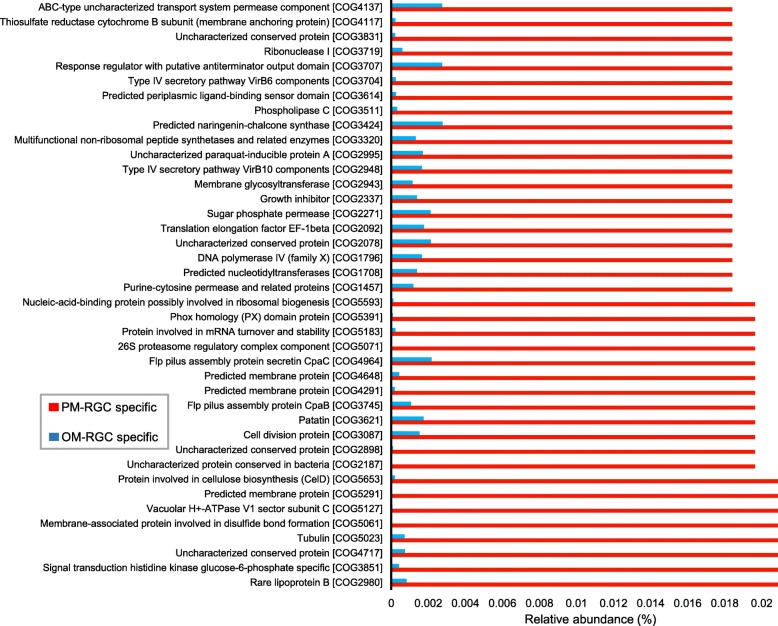
Comparison between the PM-RGC specific and OM-RGC specific ortholog functions based on the COG functions. Relative abundance of a given COG is the number of orthologs classified into this COG divided by the total number of orthologs. The 40 most abundant COGs significantly (*p* value < 0.01) enriched in PM-RGC are shown

The OM-RGC and PM-RGC specific orthologs were annotated using the COG database and compared with each other. While the PM-RGC orthologs received 3650 hits in the COG database, the OM-RGC orthologs had 4711 COG hits. Statistical analysis, based on the COG relative abundances (proportion of each COG in the total number of COGs), revealed that 2921 had significantly changed COGs (chi-squared test, *p* value < 0.05). The 40 most abundant COGs significantly enriched in PM-RGC are shown in Fig. 5. These COGs included functions for lipid and sugar metabolism, such as rarelipoprotein B (COG2980), CelD involved in cellulose biosynthesis (COG5653), and sugar phosphate permease (COG2271). Functions for cell membrane biosynthesis, such as the membrane proteins COG4291 and COG4648 without known functions, and membrane proteins involved in dissulfite bond formation (COG5061), were enriched in PM-RGC.

The 16S miTags extracted from the *Tara* Oceans metagenomes mapped to a total of 35,650 OTUs, with the rate of new gene detection at 0.01% by the end of sampling [1]. However, by sampling both surface and deep polar seawater, a large reservoir of microbial OTUs and functional genes were found that did not overlap with the *Tara* Oceans datasets. Overall, analyses of the polar seawater metagenomes increased the previously reported microbial diversity by more than 20%. Of the protein-coding genes derived from the polar microbiomes, between 20 and 40% showed a similarity with the OM-RGC generated by the *Tara* Oceans datasets. More importantly, 97% of the PM-RGC specific orthologs have unknown functions, and to our knowledge this is the first time to evaluate the overall functional specificity of polar seawater microta based on global seawater datasets. These findings suggest that there is an underestimation of the microbial taxonomic and functional diversity in the global oceans and the existence of specific and function-unknown genes.

### Microbes enriched in polar microbiomes and their genomic features

**Fig. 6:**
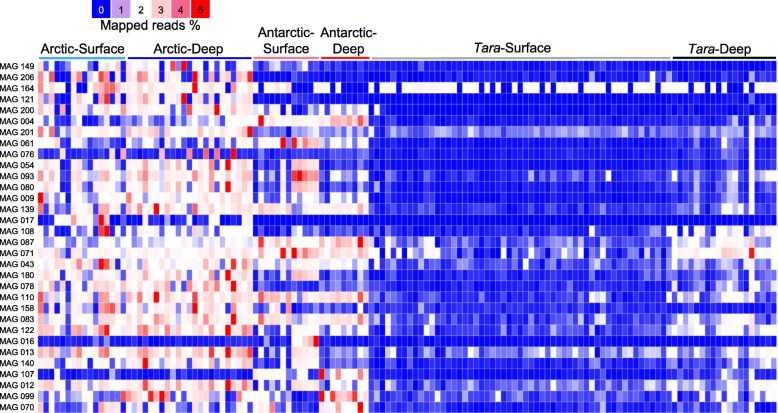
The microbes enriched in the polar seawater based on their abundance across polar and *Tara* Oceans seawater metagenomes. The abundance was calculated by mapping metagenomic reads (100,000 reads per metagenome) to single bacterial MAGs. The 32 microbes enriched for more than 50 folds (the average value of genome coverage in polar seawater metagenmes/the average value of genome coverage in *Tara* seawater metagenomes after contigs with rRNA genes masked). Taxonomic affiliations of the MAGs are listed in Additional file 6: Table S5

To further explore novel functional potential of the polar microbiota through genomic analysis, 214 microbial metagenome assembled genomes (MAGs) (> 80% completeness and < 2% potential contamination) from the 60 polar metagenomes were recovered. These microbes belonged to 24 different microbial, including Alpha-, Beta-, Gamma-, and Deltaproteobacteria, Bacteroidetes, Actinobacteria, Chloroflexi, Verrucomicrobia, and Parcubacteria (Additional file 1: Figure S2). The distribution of these 214 microbes in polar and non-polar locations was further examined by mapping reads (10 million reads per metagenome) from the 60 polar metagenomes and the 74 *Tara* Oceans metagenomes; this led to the discovery of 32 microbes enriched in polar locations (average coverage in polar metagenomes was > 50 fold higher than that in *Tara* metagenomes) (Fig. 6). The microbes enriched in polar metagenomes included members of Alpha- and Gammaproteobacteria, Actinobacteria, Bacteroidetes, Chlamydiae, and Parcubacteria; notably, some taxa (e.g., *Parcubacterium* sp. Arctic_04) were widespread in most of the polar seawater metagenomes but were almost undetectable in the *Tara* Oceans metagenomes; moreover, within the *Tara* Oceans metagenomes, these MAGs tended to be enriched in the deep ocean and almost absent in the surface seawater metagenomes (Fig. 6).

**Fig. 7:**
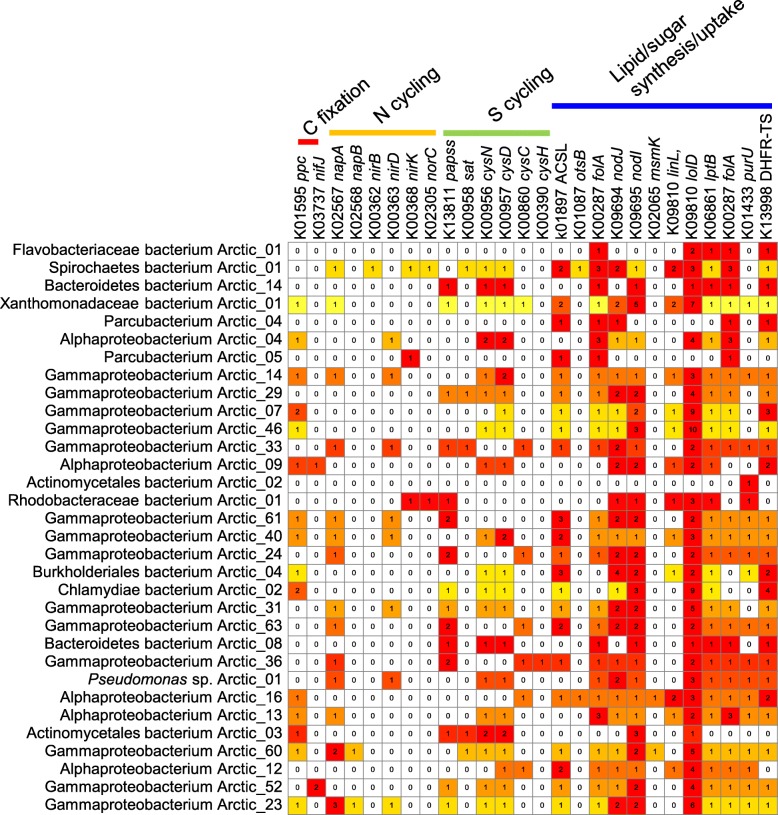
Carbon fixation, nitrogen cycling, sulfur cycling, and lipid/sugar synthetic genes in the 32 microbes enriched in polar seawater. The genes were annotated by BLASTp searching against the KEGG database

The metabolic pathways of the 32 genomes enriched in polar metagenomes were reconstructed. Based on the KEGG pathway annotation (Fig. 7), several of these genome possessed pathways for nitrogen and sulfur cycling, such as periplasmic nitrate reductase NapA (K02567) and cytochrome c-type protein NapB (K02568) for nitrate reduction, nitrite reductase large subunit nitrite NirB (K00362) and small subunit nitrite NirD (K00363) for nitrite reduction, and sulfate adenylyltransferase subunit 1 CysN (K00956) and subunit 2 CysD (K00957) for sulfate reduction. The C4-dicarboxylic acid pathway was identified as the major pathway adopted by these microbes for carbon fixation. A number of genes for sugar and lipid biosynthesis and uptake were identified in most of the genomes, such as trehalose 6-phosphate phosphatase OtsB (K01087) for trehalose biosynthesis and lipoprotein-releasing system permease proteins (K09808 and K09810) for lipid biosynthesis.

Metagenomic and gneomic analyses in the present study have revealed the functional basis for environmental filtering of microbiota in the polar seawater. Saccharide and lipid biosynthesis genes, enriched in the polar metagenomes and MAGs, can be used by microbes to combat cold environments. For example, after a temperature decrease from 37 to 16 °C (“cold shock”), trehalose levels in *Escherichia coli* cells increased up to 8-fold [30]; biosynthesis of unsaturated fatty acids, which causes a decrease in membrane fluidity of bacterial cells, is crucial to the survival of microbes at low temperature [31–33]. The synthesized polysaccharides can be components of the bacterial cell membrane [34]; consistent with the result that many membrane synthesis related genes are enriched in the polar microbiomes, suggesting their roles in cold adaptation. The C4-dicarboxylic acid pathway also occurs with high frequency in the polar microbes; this pathway does not have as high a demand for coenzymes and metals as other carbon fixation pathways [35] and this may contribute to the success of these microbes in polar regions. In addition, the occurrence of several nitrate and sulfate reduction pathways in certain microbial genomes might be a strategy of metabolic versatility to adapt to environmental change.

### Comparison between the Arctic and Antarctic microbiota

In total, 10,754 OTUs were only present in the Arctic microbiota, while 3034 OTUs were only present in the Antarctic microbiota (Additional file 1: Figure S3A). Dereplication of all the ORFs derived from the assembled Arctic metagenomes resulted in 11,806,833 orthologs, and all the assembled Antarctic metagenomes had 6,460,206 orthologs. BLASTp searching indicated that 3,268,783 (27.69%) and 1,160,189 (17.96%) orthologs are unique for the Arctic and Antarctic microbiota, respectively (Additional file 1: Figure S3B and S3C). After annotation, 4170 and 2125 COGs were identified in the Arctic specific and Antarctic specific microbiomes, respectively, and statistical analysis, based on the COG relative abundances, revealed that the two polar regions were enriched with different functions (Additional file 1: Figure S4 and Figure S5). For example, functions related to antibiotic resistance, including vancomycin resistance genes (COG2720), lantibiotic modifying enzyme (COG4403) and exporter of polyketide antibiotics (COG3559) were largely enriched in the Arctic specific orthologs (Additional file 1: Figure S4), whereas functions related to DNA recombination, DNA splicing and RNA transcription were enriched in the Antarctic specific orthologs (Additional file 1: Figure S5).

Despite the notable differences between the polar and temperate microbiomes, implying the existence of microbes that are adapted to the polar environments, comparison between the Arctic and Antarctic microbiomes identified considerable differences in terms of both taxonomy and function. This is consistent with a previous estimation that 78% of OTUs are unique to the Southern Ocean and 70% unique to the Arctic Ocean [9]. While more than 50% of the Arctic OTUs could not be found in the Antarctic microbiomes, only 27.69% orthologs are specific for the Antarctic microbiomes. This suggests that the Arctic and Antarctic microbiomes are more similar to each other at the functional level than from a taxonomic perspective. A further comparison suggests that antibiotic biosynthesis, DNA recombination, and DNA splicing are likely to be functions that contribute to the differences between Arctic and Antarctic microbiomes. The greater abundance of antibiotic resistance genes in the Arctic microbiomes might be a result of biological impacts, including human activity, which has already influenced environmental change in the Arctic [36]. DNA recombination and splicing are related to the repair of damaged DNA [37], which can be caused by exposure to ultraviolet radiation; this may be a molecular response to environmental change [38].

## Conclusions

We demonstrate microbial diversity and functional potential found in the polar microbiota and lay a foundation for studying the ecology of global ocean microbiota from a more complete perspective. We propose that environmental filtering and microbial dispersal are the major factors shaping the species composition of polar microbiota. Furthermore, we construct here the first functional gene catalog of the polar seawater microbiota, and imply that cold adaptation and environmental change are the major functional basis for microbial community assmebly at the poles.

## Materials and methods

### Sampling, DNA extraction, and metagenomic sequencing

Seawater samples were collected and filtered through 0.22-μm polycarbonate membrane filters (Millipore, MA, USA). The filters were stored in 5 mL of DNA storage buffer (500 mM NaCl, 50 mM Tris-HCl, 40 mM EDTA, and 50 mM glucose) at − 80 °C. The TIANamp Genomic DNA Kit (Tiangen Biotech, Beijing, China) was used to extract DNA from the seawater samples following the manufacturer’s protocol.

### Metagenomic quality control and assembly

Quality control of the paired-end reads was performed on a local computer server using the IlluQC.pl script documented in the software NGS QC Toolkit version 2.0 [39]. Reads containing adaptors, poor quality reads (a quality score < 20 for > 30% of the read length) or unpaired reads were removed. After quality control, the metagenomic reads (2,711,430,516 clean reads with 45,190,509 ± 9,900,733 clean reads per metagenome) were assembled into contigs (metagenomes were assembled individually) using the software MEGAHIT version 1.0.2 [40] with kmer values with kmer values from 21 to 121. The OM-RGC datasets, 55 metagenomes of surface seawater and 19 metagenomes of deep seawater in the *Tara* Oceans project [1] (http://ocean-microbiome.embl.de/companion.html) were downloaded for comparison. Detailed information about samples and corresponding metagenomic datasets is shown in Additional file 2: Table S1. Information on assembled contigs of polar seawater is shown in Additional file 3: Table S2.

### Taxonomic profiling

16S miTags were recovered from the unassembled metagenomic reads using Parallel-META3 [41]. A HMMER version 3.1 [42] search program documented in Parallel-META3 was used to predict the 16S miTags sequences from both the forward and reverse sequences. The 16S miTags were mapped to a database that integrates GreenGenes [43] with RDP [44] and SILVA [45] using the software Bowtie2 [46] and contains OTUs picked at 97% similarity or above. To reduce the false discovery rate due to sequencing errors, in a metagenome, only OTUs with two or above read numbers were returned (thus all singletons were removed). Phylum (class for Proteobacteria) and genus profiles were generated for the 60 polar seawater metagenomes using the OTU-taxa map documented in Parallel-META3 (PM-pipeline). The full list of phyla (class for Proteobacteria) and genera based on 16S miTags are given in Additional file 4: Table S3 and Additional file 5: Table S4, respectively.

### Venn analysis

The overlapped OTUs between the polar microbiomes and *Tara* Oceans microbiomes (*Tara* Oceans miTags includes 16S rRNA gene sequences extracted from 243 metagenomes) were calculated using the online Venn program (http://bioinformatics.psb.ugent.be/webtools/Venn/), where the lists of OTU identities were uploaded as input. The polar and *Tara* Oceans seawater specificity was calculated based on the formulas:

$$ {\displaystyle \begin{array}{c} Polar\ specificity=\frac{Number\ of\ polar- specific\ OTUs}{Number\ of\ OTUs\ in\ polar\ and\ Tara\ samples}\\ {} Tara\ specificity=\frac{Number\ of\ Tara- specific\ OTUs}{Number\ of\ OTUs\ in\ polar\ and\ Tara\ samples}\end{array}} $$

### Alpha- and beta-diversity analyses

Alpha- and beta-diversity analyses were performed on the polar and *Tara* Oceans seawater metagenomes using normalized data sets. 10,000 16S miTags were extracted from each metagenome using the software Seqtk (https://github.com/lh3/seqtk). The following OTUs classification was performed as described above. Chao1, Shannon, and Simpson diversity were calculated using the alpha_diversity.py script implemented in QIIME [47]. Jaccard distances, which are based on the presence/absence of OTUs, were generated using the OTU table for analyses of the community similarities, and visualized by PCoA (transformation exponent = 2) implemented in the software PAST version 2.0 [48].

### Functional analyses of the polar seawater microbiota

ORFs were predicted from the assembled contigs using MetaGeneMark version 2.8 (genes on both strands were predicted; gene overlaps were allowed; cutoff in probability of initiation and termination in non-coding state was 0.5). Protein sequences derived from the 60 polar seawater metagenomes were combined and subjected to CD-HIT (> 95% sequence identity) to generate a nonredundant catalog called PM-RGC. The PM-RGC orthologs sequences (minimum length 100) were BLASTp (*e* value < 1e-7; > 60% sequence identity for > 60% of the length of the query sequences; maximum hit number = 1) searched against the OM-RGC [1] for PM-RGC specific orthologs. To evaluate the percentage of known proteins, the PM-RGC orthologs were DIAMOND [49] BLASTp (*e* value < 1e-7; > 60% sequence identity for > 60% of the length of the query sequences; maximum hit number = 1) searched against the NCBI-Nr database. For functional annotation and classification, the PM-RGC specific and OM-RGC specific orthologs were BLASTp (*e* value < 1e-7; > 60% sequence identity for > 60% of the length of the query sequences; maximum hit number = 1) searched against the COG database [50]. Statitical analyses were performed using chi-squared test (*p* value < 0.05 as significance cutoff).

### Genome binning and analyses

Genome binning was performed with contigs using the software MaxBin2 [51] and MetaBAT [52]. To generate coverage profiles for the assembled contigs, reads from a single metagenome were mapped to the corresponding assembly using Bowtie2 (input reads were in fastq format; the script bowtie2-build was used). The output files were visualized in SAMtools version 1.10 [53] (input files were in SAM format; the scripts view, sort, and depth were used). The coverage profiles and assembled contigs were subject to the binning software MaxBin2 [51] (minimum probability for algorithm 0.8; 107 marker genes) to separate the contigs. The resulting contig groups were subjected to a second binning process using MetaBAT [52] for further separation to get MAGs. The output MAGs were checked for completeness and contamination using CheckM (lineage_wf) [54]. All the MAGs were compared for the average nucleotide identity using pyani [55] (ANIb model), and MAGs with an ANI value higher than 0.99 were considered duplicates. Taxanomy designation of the MAGs was performed using using the software GTDB-Tk v0.3.2 [56] (the script classify_wf was used). Information of the MAGs is presented in Additional file 6: Table S5.

For genome annotation, ORFs were predicted using Prodigal (single genome model; close end) and then BLASTp searched against the KEGG database (*e* value < 1e-7; > 60% sequence identity for > 60% of the length of the query sequences). Metabolic pathways in a single bacterium were reconstructed using the online tool KEGG Mapper (https://www.genome.jp/kegg/mapper.html). Genes under the categories carbohydrate metabolism and energy production were analyzed. The rRNA genes in the MAGs were predicted using the software Barrnap (https://github.com/tseemann/barrnap), under both bacteria and archaea models. The coverage profiles of the MAGs in metagenomes were calculated by Bowtie2 and SAMtools, after masking the contigs with rRNA genes.

### Data availability

All metagenomic datasets and gene catalog of PM-RGC have been deposited in the NCBI database (BioProject accession no. PRJNA588686). The 214 MAGs have been uploaded to figshare (https://figshare.com/s/fd5f60b5da7a63aaa74b) and Genbank of NCBI (BioProject accession no. SUB7116349). The PM-RGC catalog can also be downloaded at https://figshare.com/s/28fbf48ffadfade8a77f, https://figshare.com/s/2f8d1ce9fb2d68f76bb2, and https://figshare.com/s/28fbf48ffadfade8a77f.

## Supplementary information

**Additional file 1: Figure S1.** Genus-level composition based on analysis of 16S miTags. Abundant genera (the top 30 genera in terms of maximum relative abundance) are shown with all other genera grouped together as ‘Minor or unclassified’. The four groups: Arctic-Surface (0-100 m), Arctic-Deep (200-4000 m), Antarctic-Surface (0-100 m), and Antarctic-Deep (200-4000 m). **Figure S2.** Taxonomic distribution of the 214 MAGs recovered from the polar metagenomes. All the MAGs have 80% or higher completeness. **Figure S3.** Comparison between the Arctic and Antarctic microbiomes. A Venn diagram showing the distribution of OTUs across the Arctic and the Antarctic miTags. Only OTUs with more than 2 miTag numbers are included for analysis. B BLASTp searching Antarctic orthologs using the Arctic orthologs as queries. C BLASTp searching Arctic orthologs using the Antarctic orthologs as queries. Orthologs of lower than 40% similarity or higher than 1e-7 e-value were considered as ‘specific’. **Figure S4.** Functions enriched in the Arctic microbiomes. The Arctic and Antarctic-specific orthologs were annotated by searching against the COG database. Relative abundance of a given COG is the number of orthologs classified into this COG divided by the total number of orthologs. The 40 most abundant COGs significantly (p-value <0.01) enriched in the Arctic are shown. Orange color indicates antibiotic resistance genes. **Figure S5.** Functions enriched in the Antarctic microbiomes. The Arctic and Antarctic-specific orthologs were annotated by searching against the COG database. Relative abundance of a given COG is the number of orthologs classified into this COG divided by the total number of orthologs. The 40 most abundant COGs significantly (p-value <0.01) enriched in the Antarctic are shown. Purple color indicates genes involved in DNA recombination.

**Additional file 2: Table S1.** Sampling locations and metagenomes information.

**Additional file 3: Table S2.** Information of the assembled contigs.

**Additional file 4: Table S3.** Full phyla (class for Proteobacteria) list and their abundance in the polar seawater.

**Additional file 5: Table S4.** Full list of genera and their abundance in the polar seawater.

**Additional file 6: Table S5.** Information of the MAGs extracted from polar seawater metagenomes.

## Abbreviations

OM-RGC: Ocean microbial reference gene catalog
OTUs: Operational taxonomic units
PCoA: Principal coordinates analysis
PM-RGC: Polar marine reference gene catalog
