# Correction to: Structure and function of the Arctic and Antarctic marine microbiota as revealed by metagenomics

**DOI:** 10.1186/s40168-020-00871-4

**Published:** 2020-06-01

**Authors:** Shunan Cao, Weipeng Zhang, Wei Ding, Meng Wang, Shen Fan, Bo Yang, Andrew Mcminn, Min Wang, Bin-bin Xie, Qi-Long Qin, Xiu-Lan Chen, Jianfeng He, Yu-Zhong Zhang

**Affiliations:** 1 The Key Laboratory for Polar Science MNR, Polar Research Institute of China, Shanghai, 200136 China; 2 College of Marine Life Sciences, and Frontiers Science Center for Deep Ocean Multispheres and Earth System, Ocean University of China, Qingdao, 266003 China; 3 State Key Laboratory of Microbial Technology, and Marine Biotechnology Research Center, Shandong University, Qingdao, 266237 China

**Correction to: Microbiome (2020) 8:47**

**https://doi.org/10.1186/s40168-020-00826-9**

Following publication of the original article [1], the authors reported an error in the order of author list and the Funding section. The correct author list and the updated Funding section are included in this correction, and the original article has been corrected.

**Funding**

This work was funded by the National Key R & D Program of China (2018YFC1406700), the Scientific Research Staring Foundations of Ocean University of China (841912034 and 841912035), The response and feedback of the Southern Ocean to climate change (RFSOCC2020–2025), the National Science Foundation of China (41976230, 41906201, 41476168, U1706207, 31630012, 91851205, 31670063), the Major Scientific and Technological Innovation Project of Shandong Province (2019JZZY010817), the Program of Shandong for Taishan Scholars (tspd20181203), and AoShan Talents Cultivation Program Supported by Qingdao National Laboratory for Marine Science and Technology (2017ASTCP-OS14).
