# Supplementary material for: Structure and function of the Arctic and Antarctic marine microbiota as revealed by metagenomics
Source: Microbiome. 2020 Apr 2;8:47. doi: 10.1186/s40168-020-00826-9 (PMC7119284; doi:10.1186/s40168-020-00826-9)
Supplement: Supplementary file 2 — Additional file 1: Figure S1. Genus-level composition based on analysis of 16S miTags. Abundant genera (the top 30 genera in terms of maximum relative abundance) are shown with all other genera grouped together as ‘Minor or unclassified’. The four groups: Arctic-Surface (0-100 m), Arctic-Deep (200-4000 m), Antarctic-Surface (0-100 m), and Antarctic-Deep (200-4000 m). Figure S2. Taxonomic distribution of the 214 MAGs recovered from the polar metagenomes. All the MAGs have 80% or higher completeness. Figure S3. Comparison between the Arctic and Antarctic microbiomes. A Venn diagram showing the distribution of OTUs across the Arctic and the Antarctic miTags. Only OTUs with more than 2 miTag numbers are included for analysis. B BLASTp searching Antarctic orthologs using the Arctic orthologs as queries. C BLASTp searching Arctic orthologs using the Antarctic orthologs as queries. Orthologs of lower than 40% similarity or higher than 1e-7 e-value were considered as ‘specific’. Figure S4. Functions enriched in the Arctic microbiomes. The Arctic and Antarctic-specific orthologs were annotated by searching against the COG database. Relative abundance of a given COG is the number of orthologs classified into this COG divided by the total number of orthologs. The 40 most abundant COGs significantly (p-value <0.01) enriched in the Arctic are shown. Orange color indicates antibiotic resistance genes. Figure S5. Functions enriched in the Antarctic microbiomes. The Arctic and Antarctic-specific orthologs were annotated by searching against the COG database. Relative abundance of a given COG is the number of orthologs classified into this COG divided by the total number of orthologs. The 40 most abundant COGs significantly (p-value <0.01) enriched in the Antarctic are shown. Purple color indicates genes involved in DNA recombination. [file 40168_2020_826_MOESM1_ESM.docx]

Supplementary Figures for

**Structure and Function of the Arctic and Antarctic Marine Microbiota as Revealed by Metagenomics**

**Figure S1** Genus-level composition based on analysis of 16S miTags. Abundant genera (the top 30 genera in terms of maximum relative abundance) are shown with all other genera grouped together as ‘Minor or unclassified’. The four groups: Arctic-Surface (0-100m), Arctic-Deep (200-4,000m), Antarctic-Surface (0-100m), and Antarctic-Deep (200-4,000m).

**Figure S2** Taxonomic distribution of the 214 MAGs recovered from the polar metagenomes. All the MAGs have 80% or higher completeness.

**Figure S3** Comparison between the Arctic and Antarctic microbiomes. **A** Venn diagram showing the distribution of OTUs across the Arctic and the Antarctic miTags. Only OTUs with more than 2 miTag numbers are included for analysis. **B** BLASTp searching Antarctic orthologs using the Arctic orthologs as queries. **C** BLASTp searching Arctic orthologs using the Antarctic orthologs as queries. Orthologs of lower than 40% similarity or higher than 1e-7 e-value were considered as ‘specific’.

**Figure S4** Functions enriched in the Arctic microbiomes. The Arctic and Antarctic-specific orthologs were annotated by searching against the COG database. Relative abundance of a given COG is the number of orthologs classified into this COG divided by the total number of orthologs. The 40 most abundant COGs significantly (p-value < 0.01) enriched in the Arctic are shown. Orange color indicates antibiotic resistance genes.

**Figure S5** Functions enriched in the Antarctic microbiomes. The Arctic and Antarctic-specific orthologs were annotated by searching against the COG database. Relative abundance of a given COG is the number of orthologs classified into this COG divided by the total number of orthologs. The 40 most abundant COGs significantly (p-value < 0.01) enriched in the Antarctic are shown. Purple color indicates genes involved in DNA recombination.
